# Correlation between skeletal muscle fiber type and responses of a taste sensing system in various beef samples

**DOI:** 10.1111/asj.13425

**Published:** 2020-07-21

**Authors:** Yusuke Komiya, Wataru Mizunoya, Kurumi Kajiwara, Issei Yokoyama, Hideki Ogasawara, Keizo Arihara

**Affiliations:** ^1^ Department of Animal Science School of Veterinary Medicine Kitasato University Towada Japan; ^2^ Department of Animal Science and Biotechnology School of Veterinary Medicine Azabu University Sagamihara Japan; ^3^ Field Science Center School of Veterinary Medicine Kitasato University Yakumo Japan

**Keywords:** meat, muscle fiber type, MyHC1, slow‐twitch muscle, taste sensor

## Abstract

The difference of muscle fiber type composition affects several parameters related to meat quality; however, the relationship between muscle fiber types and meat taste is unclear. To elucidate this relationship, we determined the taste of various beef samples using a taste sensor (INSENT SA402B) and analyzed its correlation with different muscle fiber type composition. We used 22 kinds of beef samples and measured nine tastes, including the relative and change of membrane potential caused by adsorption (CPA) values, using six sensors (GL1, CT0, CA0, AAE, C00, and AE1). The taste sensor analysis indicated positive value outputs for the relative C00, AAE, and GL1 values as well as for the CPA value of AAE, which corresponded to bitterness, umami, sweetness, and richness, respectively. We found significant positive correlations of the myosin heavy chain 1 (MyHC1) composition with umami taste, and with richness. This result suggests that high levels of slow MyHC1 can induce strong umami taste and richness in beef. We expect that our results will contribute to the elucidation of the relationship between muscle fiber types and meat palatability.

## 1 INTRODUCTION

Skeletal muscle tissues are composed of muscle fibers, which are classified into two main types, type 1 (slow‐twitch) and type 2 (fast‐twitch) (Schiaffino & Reggiani, 2011). Type 1 fibers are rich in mitochondria and myoglobin, have high oxidative capacity, and are resistant to fatigue. Type 2 fibers are further subdivided into types 2A, 2X, and 2B in order of increasing contraction speed. Type 2B fibers exhibit high glycolytic metabolism and fatigue easily, while type 2A and 2X fibers have intermediate characteristics between type 1 and type 2B fibers. Myosin heavy chain (MyHC) is a predominant and key component of skeletal muscle proteins and is used as a muscle fiber type biomarker. In adult mammalian skeletal muscles, four predominant MyHC isoforms have been identified; MyHC1, 2A, 2X, and 2B, which are preferentially expressed in respective fibers (Hagiwara, 2013). MyHC2B appears to be specific to small mammals (Liu et al., 2009) and marsupials (Zhong, Lucas, & Hoh, 2008). In bovine muscles, type 2B fibers are not expressed in the limb or trunk muscles (Toniolo et al., 2005). Similarly, type 2B fibers are not expressed in human muscles; however, it appears that in human muscles, type 2X fibers are considered type 2B fibers (Hagiwara, 2013).

The composition of muscle fiber type is thought to affect the color, pH, water‐holding capacity, tenderness, and nutritional value of meat (Choi & Kim, 2009; Lee, Joo, & Ryu, 2010). Taste is an important factor in evaluating meat quality; however, the relationship between taste and the composition of different meat muscle fiber types is not fully understood. Chikuni et al. (2010) reported that type 1 rich bovine muscle tissues, such as the masseter and the diaphragm, are less sour and more bitter and astringent than type 2 rich muscle tissues. However, the relationship between the difference of muscle fiber type composition and other tastes, especially the umami and sweet tastes, which are important in meat palatability, is not well known.

The free amino acid level is important for food taste (Nishimura, Ra Rhue, Okitani, & Kato, 1988). For example, the results of a sensory evaluation test showed that L‐glycine (Gly) and L‐alanine (Ala) are associated with the sweet taste, while monosodium L‐glutamate and monosodium L‐aspartate are associated with the umami taste (Kawai, Sekine‐Hayakawa, Okiyama, & Ninomiya, 2012). Moreover the total free amino acids have been characterized as umami‐related substances as described previously (Sasaki et al., 2005). Therefore, the difference in the amount and composition of free amino acids in the food could result in different tastes. A recent study revealed that the composition of MyHC1 has a strong positive correlation with the total free amino acid levels in beef (Mashima et al., 2019). Moreover among the 31 measured free amino acids and dipeptides, 11 amino acids, including Ala, showed significant positive correlations with the composition of MyHC1. Therefore, the difference of muscle fiber type composition could affect the taste of meat, such as its umami and sweet components, owing to the higher total amino acid and Ala levels.

The aim of this study was to determine the relationship between muscle fiber types and beef taste using a taste sensor (INSENT SA402B; INSENT). The taste sensor used can evaluate the taste perceived by humans and is called an electronic tongue with global selectivity, which is the property of discriminating among taste qualities and quantifying them without discriminating among the chemical substances (Wu, Tahara, Yatabe, & Toko, 2020).

## 2 MATERIALS AND METHODS

### 2.1 Meat sample preparation

We used 22 types of beef samples obtained from butcher shops (raw meat samples stored at 4°C) or collected from Japanese Shorthorn cattle. All samples were stored at −30°C immediately after obtained. Detailed information of the beef samples is shown in Table 1. Frozen meat samples (approximately 50 g each) were thawed at 4°C overnight and then were cut into 1 cm squares, with as much of their fat and connective tissue as possible removed, and homogenized in distilled water (approximately 250 ml). The homogenates were centrifuged at 3000 rpm for 30 min (4°C). The supernatant was filtered on ice and the samples were stored at −30°C until the taste sensor analysis.

**TABLE 1:** Information of beef samples

Sample ID	Meat part	Days after slaughter	Country (breed)
1	Short plate	Unknown	Australia
2	Chuck eye roll		
3	Clod		
4	Outside skirt		
5	Round		
6	Tongue		USA
7	Short plate	8 month	Japan (Japanese Black)
8	Loin	6 month	
9	Round	6 month	Japan (F1)
10	Tenderloin	8 month	
11‐16	Top round	12 or 21 month	Japan (Japanese Shorthorn)
17‐22	Rib eye roll	12 or 21 month	

F1: meat breed × dairy breed.

### 2.2 MyHC isoform composition

The determination of the MyHC isoform composition in the meat samples was performed by high‐resolution sodium dodecyl sulfate polyacrylamide gel electrophoresis (SDS‐PAGE) (Mizunoya, Wakamatsu, Tatsumi, & Ikeuchi, 2008). Briefly, frozen powdered samples (approximately 50 mg each) were homogenized in an SDS solution [10% SDS, 40 mM dithiothreitol (DTT), 5 mM ethylenediamine tetraacetic acid (EDTA), and 0.1 M Tris‐HCl buffer (pH 8.0)] that contained 1% of a protease inhibitor cocktail (Nacalai Tesque, Inc.). The sample homogenates were heated in boiling water for 3 min. The total protein concentrations were assayed using the Pierce BCA Protein Assay Kit (Thermo Fisher Scientific) and the samples were diluted in a 2 × sample buffer [100 mM DTT, 4.0% SDS, 0.16 M Tris‐HCl (pH 6.8), 43% glycerol, and 0.2% bromophenol blue] and dH_2_O to produce final protein concentrations of 10 ng/μl in a 1 × sample buffer. The separating gel was 8% acrylamide (acrylamide/bisacrylamide ratio = 99:1) and contained 35% (v/v) glycerol. After loading the samples, electrophoresis was performed at a constant voltage of 140 V for 22 hr at 4°C. The gels were stained with Silver Stain Kanto III (Kanto Chemical Co., Inc.). The bands were captured on an image scanner, and the relative MyHC isoform contents were quantified by densitometry using the ImageJ 1.50i software (Rasband, W.S., National Institutes of Health). MyHC isoforms were identified according to their different migration rates (MyHC1 > 2). A mixed sample of rat soleus and extensor digitorum longus muscles was used as the four MyHC isoform references (the migration rate was MyHC1 > 2B> 2X > 2A).

### 2.3 Taste sensor analysis

The taste sensing system SA402B (INSENT) was used to determine the tastes of the beef samples. The taste sensor imitated the human tongue, and simulated the taste perception of living organisms using artificial lipids as transducers for the multichannel taste sensors. It consisted of six lipid membrane sensor probes that distinguished among sweetness (GL1), saltiness (CT0), sourness (CA0), umami (AAE), bitterness (C00), and astringency (AE1), thereby imitating the function of the human caliculus gustatorius cells, while taste was distinguished by the pattern recognition of the potential change (Wu et al., 2020). According to the INSENT manufacturer's manual, the sensor signals of a sample are different from those of the standard solution (30 mM KCl and 0.3 mM L‐(+)‐Tartaric Acid) and convert to the value corresponding to the individual tastes (Wu et al., 2020). The measurements of the taste sensor result in two output types, a relative value and a change of membrane potential caused by adsorption (CPA) value. The CPA value expresses the difference between the potential of the reference solution before and after the sample measurement and corresponds to aftertaste. We measured nine tastes, including the relative and CPA values, using six sensors. The correspondence between the sensor outputs and the tastes is shown in Table 2. In general, the converted value 1 corresponded to a 20% difference from the standard sample in the sensory evaluation (Wu et al., 2020).

**TABLE 2:** MyHC isoform composition (%) and taste sensor output (mV) of various meat samples

Sample ID	MyHC isoform		Taste sensor output (relative value)						Taste sensor output (CPA value)		
	MyHC1	MyHC2	CA0	C00	AE1	AAE	CT0	GL1	C00	AE1	AAE
1	37.1	62.9	−26.63	2.58	−0.94	10.93	−10.50	0.72	−0.12	−0.70	2.35
2	50.9	49.1	−26.70	2.62	−1.18	10.84	−11.16	0.51	−0.05	−0.64	2.38
3	54.1	45.9	−30.01	4.48	−1.06	11.83	−10.74	3.32	0.04	−0.69	2.83
4	88.7	11.3	−33.23	5.11	−0.83	11.40	−11.88	2.10	0.06	−0.70	2.88
5	32.0	68.0	−25.06	1.75	−1.32	10.48	−11.73	−0.33	−0.18	−0.76	2.03
6	60.3	39.7	−26.71	3.18	−0.59	11.40	−10.34	1.52	−0.20	−0.27	1.39
7	39.4	60.6	−26.99	3.34	−0.35	11.58	−9.88	1.07	−0.25	−0.34	1.00
8	52.2	47.8	−25.51	3.02	−0.77	10.81	−14.25	−0.72	−0.19	−0.36	0.87
9	54.1	45.9	−22.90	1.13	−0.61	10.68	−12.04	−1.14	−0.37	−0.39	1.52
10	30.5	69.5	−23.69	1.87	−0.64	10.72	−12.77	−0.75	−0.35	−0.56	1.04
11	14.6	85.4	−19.69	2.41	−0.21	10.14	−11.68	−0.30	−0.23	−0.24	1.09
12	19.2	80.8	−19.55	2.78	−0.09	10.16	−11.68	−0.01	−0.16	−0.24	1.44
13	55.9	44.1	−21.36	2.54	−0.13	10.59	−13.44	0.68	−0.24	−0.22	0.92
14	27.5	72.5	−20.84	6.90	−0.10	10.49	−11.01	1.11	0.17	−0.20	0.93
15	26.0	74.0	−21.06	5.32	−0.31	10.51	−12.55	0.49	−0.02	−0.18	1.02
16	32.3	67.7	−20.91	7.10	−0.10	10.66	−10.93	1.26	0.19	−0.16	1.06
17	8.9	91.1	−20.18	2.52	−0.23	10.63	−11.40	0.98	−0.09	−0.26	1.05
18	22.3	77.7	−20.46	2.95	−0.37	10.90	−10.94	1.00	−0.02	−0.27	1.14
19	14.6	85.4	−21.62	3.15	−0.25	11.31	−11.72	1.70	0.01	−0.25	1.14
20	56.1	43.9	−22.01	7.86	−0.15	11.38	−10.07	2.88	0.32	−0.16	1.25
21	67.1	32.9	−21.45	7.50	−0.12	11.18	−10.50	2.50	0.35	−0.20	1.32
22	42.3	57.7	−21.94	8.15	−0.07	11.47	−10.10	3.58	0.33	−0.16	1.41

The correspondence between sensor output (relative value) and taste: CA0, sourness; C00, acidic bitterness; AE1, astringency; AAE, umami; CT0, saltiness; GL1, sweetness. The correspondence between sensor output (CPA value) and taste: C00, aftertaste from acidic bitterness; AE1, aftertaste from astrigency; AAE, richness. The correspondence between sensor outputs and tastes was followed by Kobayashi et al., (2010).

### 2.4 Statistical analysis

Pearson's correlation coefficient was calculated using Microsoft Excel (2011) to determine the association between the MyHC1 composition and each taste sensor output. A two‐tailed Student's *t* test was used to calculate the *p* values and a value of *p* < 0.05 was considered significant.

## 3 RESULTS

### 3.1 MyHC isoform composition

**FIGURE 1:**
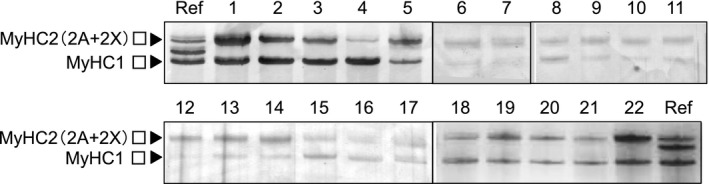
Separation of myosin heavy chain (MyHC) isoforms by sodium dodecyl‐sulfate polyacrylamide gel electrophoresis (SDS–PAGE) analysis for each muscle sample. Sample ID: 1, short plate (AUS); 2, chuck eye roll (AUS); 3, clod (AUS); 4, outside skirt (AUS); 5, round (AUS); 6, tongue (USA); 7, short plate (JB); 8, loin (JB); 9, round (JB); 10, tenderloin (JB); 11–16, top round (JSH); 17–22, ribeye roll (JSH); Ref, a mix sample of rat soleus and extensor digitorum longus muscle was used as the four MyHC isoform references (the migration rate was MyHC1>2B>2X>2A). Abbreviations: Australia, AUS; Japanese Black, JB; Japanese Shorthorn, JSH

We measured the muscle fiber type compositions in twenty‐two bovine muscle tissue samples (Figure 1). The separation of MyHC2A and 2X was not sufficient under the present electrophoretic conditions, and we used the sum of MyHC2A and 2X as the MyHC2 content. Different MyHC isoform compositions (MyHC1 and MyHC2) were observed in the various muscle tissues (Table 2). The MyHC1 was the highest (88.7%) in the outside skirt (AUS) and the lowest (8.9%) in the ribeye roll (JSH) samples.

### 3.2 Taste sensor analysis

The results of the taste sensor analysis of various meat samples are shown in Table 2. According to a previous study (Wu et al., 2020), humans can discriminate between different tastes when the concentration difference between the taste substances is higher than 20%. Thus, the difference was defined as 1 unit. The comparison of the sensor outputs for each meat sample, some of which differed by more than 1 unit, shown in Table 2, suggests that the electric taste sensing system was able to detect taste differences among the different meat samples. A negative value represented a taste that is considered imperceptible by the human gustatory system. The electric taste sensing system indicated positive values in the relative value outputs of C00, AAE, and GL1 as well as the CPA value of AAE, which corresponded to bitterness, umami, sweetness, and richness (umami aftertaste), respectively. The taste values obtained ranged from 1.13–8.15 in bitterness, 10.14–11.83 in umami, −1.14–3.58 in sweetness, and 0.87–2.88 in richness (Table 2). The rest of the sensors indicated negative values, which meant that the tastes corresponding to the sensors were not at a level that could be perceived by humans.

### 3.3 Correlation between the MyHC1 composition and the taste sensor outputs

**FIGURE 2:**
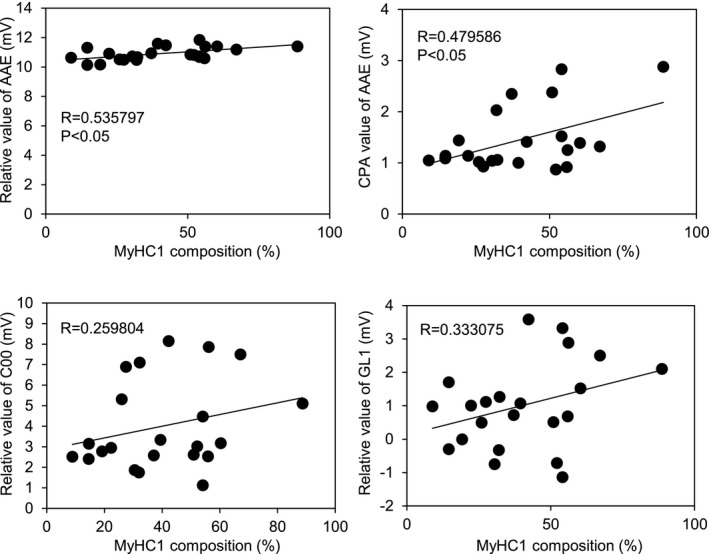
Correlation between the composition of myosin heavy chain 1 (MyHC1) and the taste sensor outputs in beef samples. The correspondence between each sensor output and each taste is described below: relative value of AAE, umami; CPA value of AAE, richness; relative value of C00, bitterness; relative value of GL1, sweetness

We investigated the correlation between the proportion of MyHC1 and the four sensor outputs (the relative values of C00, AAE, GL1, and the CPA value of AAE), which had positive values. We found a significant positive correlation of the MyHC1 proportion with the relative value of the AAE sensor, and with the CPA value of AAE sensor (*p* < .05) (Figure 2). This result suggests that the MyHC1 composition influenced the umami and richness of beef.

## 4 DISCUSSION

The association between the different muscle fiber types and certain meat characteristics has been reviewed extensively by several researchers. The difference of muscle fiber type composition is considered to affect the color, pH, water‐holding capacity, tenderness, and nutritional value of meat (Choi & Kim, 2009; Lee et al., 2010). However, the relationship between taste and different muscle fiber types is not fully understood. Therefore, we tried to elucidate this relationship by using a taste sensor.

The meat samples were subjected to six taste sensors, the output of which was nine tastes, including the relative and CPA values. As is shown in Table 2, four of the nine tastes (acidic bitterness, umami, sweetness, and richness) had positive values for most meat samples. It could be suggested that humans recognize the combination of these four tastes as the taste of meat. Chikuni et al. (2010) reported that type 1 rich bovine muscle tissues, such as the masseter and the diaphragm ones, are less sour and more bitter and astringent than type 2 rich muscle tissues, while there are no significant differences in the umami taste of different muscle fiber types. The results we obtained regarding bitterness were in agreement with the results of the aforementioned study; however, the same was not true for our results regarding the umami taste. We speculate that this difference could be attributed to the conditions of the sample treatment. Chikuni et al. (2010) used 5‐g samples of cooked minced meat (added 1% sodium chloride and heated them at 200°C for 4 min on each side) to analyze the taste sensors. We used 50‐g samples of raw meat that was not treated with heat or added seasonings. The difference of sample preparation could have been why we obtained different results from Chikuni et al. (2010). We also performed the taste sensor analysis using boiled beef samples. As a result, there was similar result in the correlation between MyHC1 and tastes, although there was a change in the sensor response value (data not shown). In future, we should try taste sensor analysis using meat samples cooked with various methods.

We found a significant positive correlation between the MyHC1 composition and two tastes (umami and richness) (Figure 2). Umami is the fifth taste discovered by Ikeda in 1909 (Ikeda, 2002). Umami is defined as the taste properties resulting from the natural occurrence or intentional addition of compounds such as monosodium glutamate and certain 5’‐nucleotides such as 5′‐inosinate and 5′‐guanylate (Maga, 1994), and richness is recognized as the aftertaste of umami. These tastes are important factors as meat taste properties. The AAE sensor was designed to respond to the umami compounds. Umami is evoked by some amino acids, therefore it could be suggested that the umami taste of meat is related to free amino acids. A sensory evaluation test performed showed that Gly, Ala, L‐serine (Ser), L‐glutamic acid (Glu), L‐asparagine (Asn), and L‐glutamine (Gln) elicited the umami taste (Kawai et al., 2012). In addition, Mashima et al. (2019) reported that 11 amino acids showed significant positive correlations with the MyHC1 composition. The amino acids that were found to have a positive correlation with the MyHC1 composition in this report were Ala, β‐Ala, Gln, histidine (His), 1‐methyl His, 3‐methyl His, hydroxyproline, ornithine, Ser, taurine, and tryptophan. The results of the aforementioned two studies indicated that Ala, Ser, and Gln, which are related to the umami taste, were positively correlated with the MyHC1 composition. Therefore, these amino acids, or their combination, could have contributed to the positive correlation between the umami taste (including richness) and the MyHC1 composition of beef obtained in our study. The slow‐twitch fiber dominant muscle had a stronger umami taste; however, there was no significant difference in the concentration of Glu, one of the most important umami substrates, among the muscle fiber types. Wang et al. (2020) reported that organic acids affect the umami taste as do amino acids and 5’‐nucleotides. Therefore, the positive correlation between the slow‐twitch muscle and the umami taste obtained in our study may have been derived by organic acids. Organic acids are important substrates of the Krebs cycle and are metabolized in the mitochondria. There are several reports about the relationship of organic acids and muscle fiber types. A slow‐twitch muscle increase results in the increase of organic acids in the Krebs cycle in the skeletal muscle of mice (Hatazawa et al., 2015). The expression levels of enzymes regulating the flux of substrates in the Krebs cycle are different in different skeletal muscle fiber types (Rakus, Gizak, Deshmukh, & Wïniewski, 2015; Schiaffino, Reggiani, Kostrominova, Mann, & Murgia, 2015). However, the direct relationship between muscle fiber types and organic acids has not been elucidated, thus it should be clarified in the future.

Bitterness is an important taste that helps prevent the intake of poisonous materials. It is often considered distasteful, however low bitterness intensity is recognized as richness. According to some reports, the threshold for quinine compounds, one of the bitter components, is 0.008 mM (Izawa, Amino, Kohmura, Ueda, & Kuroda, 2010) and the taste sensor output of the 0.01 mM quinine compound is approximately 10 (Kobayashi et al., 2010). In our experiments, the maximum output indicated by the bitterness sensor was 8.15, which is not supposed to be perceived as bitterness by humans. As the values obtained in our study were not meant to be perceived as bitterness, we speculated that the detection of bitterness indicated the richness of the meat.

Sweetness, which is produced by sugars or sugar alcohols, has a role in indicating nutrient sources. To the best of our knowledge, this is the first report on the evaluation of beef sweetness using taste sensors. The electric taste sensing system indicated positive values in the GL1 sensor outputs, which suggested that sweetness can be detected in meat samples. In general, the GL1 taste sensor can quantify the sweetness of uncharged sweeteners, such as glucose, sucrose, and fructose (Wu et al., 2020). The aging process of meat generates a variety of taste compounds (Nishimura, 1998). It is thought that most of the monosaccharides are lost in meat owing to the anaerobic glycolysis system caused by postmortem changes. However, it has been suggested that the glycogen breakdown in muscle meat after slaughter, does not only generate lactic acid, but also monosaccharides such as glucose. Rhoades et al. (2005) reported that a small amount of glucose was present in beef 4 days after slaughter. In addition to glycogen, the degradation of glycosphingolipids in beef (Fong et al., 2016) or proteoglycans in intramuscular connective tissue (Nishimura, 2010) may also be related to the monosaccharide increase, however, the details of this topic are not clear. The results obtained in our study could indicate that the sweetness sensor detected degradation products derived from glyco‐related compounds in meat.

The other five tastes had mostly negative values, indicating that they cannot be perceived in meat by humans, as a negative value represented a taste that is considered imperceptible by the human gustatory system.

## 5 CONCLUSION

Meat palatability is determined by many factors, including taste, flavor, texture, temperature, color, and experience with food. In this study, we found a significant positive correlation between the composition of MyHC1 and taste, especially umami and richness, in beef samples. According to the results of previous studies, these results could be attributed to the free amino acids in beef (Mashima et al., 2019). These results suggest that high levels of slow MyHC1 possibly induce a strong umami taste and richness in bovine muscles high in free amino acids. In future, we would like to clarify the relationship between proportion of slow‐twitch muscle and the palatability of meat, in combination with other factors (color, pH, water‐holding capacity, tenderness, and flavor), as meat palatability is not determined by taste alone.
